# Uncommon Affections of the Eyelids

**Published:** 1897-04

**Authors:** J. Hobart Egbert

**Affiliations:** Holyoke, Mass.


### UNCOMMON AFFECTIONS OF THE EYELIDS.

BY J. HOBART EGBERT, A. M., M. D., PH. D., HOLYOKE, MASS.

I. PRIMARY VACCINE PUSTULE ON EYELID.—A short time ago, Mary A., eleven years old, came to the dispensary for treatment of a troublesome sore on her eyelid. The conditions presented were: An elevated ulcer, pustular in character, on the free margin of the right lower lid about half an inch from the external canthus; epiphora, swelling of eyelid and injection of conjunctiva. The ulcer extended about one-fourth of an inch along the ciliary margin, was about one eighth of an inch wide, had a depressed center, and the cuticular surface adjacent to it was red and swollen. The patient complained of ocular and frontal pain. There was mild systemic disturbance, slight coating of the tongue, headache, and slight elevation of temperature. According to the history obtained, a small pimple had appeared on the lid four or five days previously, had "festered" and, although frequently bathed with warm water, had continued to enlarge until the conditions just described obtained. The child thought she had scratched her eyelid, about a week before, at or near the same place now occupied by the ulcer, but it had apparently amounted to nothing. The appearance of the lesion at once suggested vaccine pustule. Upon inquiry it was ascertained that the patient had not been vaccinated since infancy, but that her little sister had been vaccinated about two weeks previously, and had had a very sore arm. The patient and the sister usually occupied the same bed. The diagnosis of "accidental vaccination of the eyelid" was made, the eye washed with a warm aqueous solution of boric acid, the lesion dusted with calomel, and yellow oxid ointment (yellow mercury oxid, 2 grains; vaselin, ½ ounce) prescribed for local application. A saline laxative was also prescribed and rest within-doors enjoined. Under this treatment the inflammation gradually subsided, and in about two weeks' time the lesion healed, leaving only a slight marginal scar on the eyelid with the permanent loss of four or five eyelashes.

Rutherford (*Quar. Med. Jour.* iv., part 4) recently reported a case in which a mother was inoculated in the eye and on the eyelids by contact with the pustule occasioned by the vaccination of her four-months-old child. According to the report, the patient recovered without serious permanent injury.

II. PRIMARY SYPHILITIC LESION ON EYELID.—The following rare case—rare at least in its origin—came under observation somewhat more than a year ago. The patient, George N., a negro boy, five years old, presented, when first seen, the following conditions: At the external canthus of the left eye and including the outer portion of the free margin of the lower eyelid, was a small ulcer, having a concave surface coated with yellowish exudate; its outline was quite regular, and its edges were indurated and elevated above the surrounding tissue; both ocular and palpebral conjunctivæ were deeply injected, there was more or less epiphora, some photophobia, but evidently little local pain. The child appeared rugged and well-nourished, and the teeth gave no indication of hereditary disease. The resemblance of the lesion to a primary syphilitic sore was at once observed, but a satisfactory history was not easily obtained. The father was said to be well, or "quite so," and the same was said of the rest of the family.

From the information given it was ascertained that the sore had been "coming" on the child's eye for three or four weeks. Further examination of the patient revealed the fact that the post-cervical glands of the neck were somewhat swollen, while the left parotid gland was somewhat sensitive to pressure. The lesion was treated with a 2 per cent. solution of silver nitrate; $\frac{1}{10}$ grain of calomel, in tablet form, was ordered to be given after each meal, and an antiseptic wash and ointment of yellow mercury oxide prescribed for local use. This case was kept under observation for some time, and proved to be syphilis. In due time mucous patches appeared in the throat and the body was covered with a papular eruption. The lesion on the eyelid disappeared in regular order, and the boy is now apparently restored to health, although advice has been given to continue the anti-syphilitic treatment for some time to come. How the chancre came to be situated on the eyelid has never been satisfactorily explained.

III. "HORN" OF THE EYELID.—In the year 1892, Mr. J. S., aged 59, a resident of Polk Co., Missouri, consulted the writer concerning a troublesome appendage which was growing upon the lower lid of his right eye. The growth proved to be a cutaneous horn, nearly one inch and a quarter long, rounded at its free extremity and flattened toward the base. It was attached to the ciliary margin of the eyelid slightly external to its center and caused the lid to hang down in such manner as to expose its conjunctival surface. As a result of this eversion of the lid, the tears ran out upon the cheek, nictitation was rendered difficult, and both ocular and palpebral conjunctivæ were in a state of chronic inflammation. The growth had commenced as a small wart upon the margin of the lid and had reached this extensive development in less than two years' time. The horn was successfully removed by making a V-shaped incision about its base and closing with sutures the artificial coloboma of the lid thus formed. The conjunctivitis was then easily controlled and a radical cure effected. A similar case was reported by Dr. Henry Shaw, of Boston, in the *Boston Medical and Surgical Journal*, for February 11, 1869.—*Phila. Polyclinic.*

